# Cardiac protective techniques in left breast radiotherapy: rapid selection criteria for routine clinical decision making

**DOI:** 10.1186/s40001-023-01470-3

**Published:** 2023-11-09

**Authors:** Meltem Kirli Bolukbas, Sibel Karaca, Volkan Coskun, Esengul Kocak Uzel

**Affiliations:** 1 Department of Radiation Oncology, Health Sciences University Bakirkoy Dr. Sadi Konuk Training and Research Hospital, Tevfik Saglam Street, Bakirkoy, 34140 Istanbul, Turkey; 2 Department of Radiation Oncology, Akdeniz University, Dumlupinar Boulevard, Pınarbası Konyaalti, Antalya, Turkey

**Keywords:** Breast cancer, Cardiac protective techniques, Heart-sparing RT, Dosimetric effects, Selection criteria

## Abstract

**Objective:**

In left breast radiotherapy (RT) desired heart doses may be achieved without heart-sparing RT techniques in some patients. We aimed to examine the existence of predictive factors and cutoff points to determine which patients are the main candidates for heart-sparing RT techniques.

**Material and method:**

Dosimetric data for left breast cancer was examined. RT plans were made at conventional doses to the breast and peripheral lymph nodes. Statistical analyses were performed using SPSS 22.0 (SPSS Inc., IBM Corp., Armonk, NY).

**Result:**

114 cases were evaluated by ROC (Receiver operating characteristic) analysis in the breast-conserving surgery (BCS) and mastectomy groups. While only left lung volume (AUC: 0.74, 95% CI 0.61–0.87, *p* = 0.002) was significant in BCS cases, in cases with mastectomy, left lung volume (AUC: 0.81, 95% CI 0.69–0.94, *p* = 0.002) and lung/heart volume ratio (AUC: 0.83, 95% CI 0.70–0.96, *p* = 0.001) had a significant relationship with the relevance of heart doses. The cutoff point of 1.92 was selected for the lung/heart volume ratio for the mastectomized patients. Moreover, the cutoff point 1154 cc and 1208 cc was determined for the left lung volume for the BCS and mastectomized patients, respectively.

**Conclusion:**

Various cutoff points in left breast RT can be used to predict whether RT plans will meet QUANTEC (Quantitative Analysis of Normal Tissue Effects in the Clinic) heart dose limits. Evaluating only these few cutoff points before planning makes it possible to eliminate 70% of patients with BCS and 40% of patients with mastectomy from respiratory-controlled methods, which require time and effort. Patients with lung volume and lung/heart volume ratio smaller than the cutoff values can be considered primary candidates for heart-sparing techniques.

## Introductıon

Breast radiation therapy (RT) is an indispensable part of treating breast cancer. Local control and overall survival increase with RT. This situation makes RT a crucial component of adjuvant therapy in both BCS (breast-conserving surgery) cases and most mastectomy cases [1–4]. With the increase in survival, long-term radiation toxicity becomes a major concern. The heart is the most vital organ at risk (OAR) in left breast cancer RT. Numerous large-sampled studies with breast cancer patients indicate a relationship between cardiac mortality/toxicity and cardiac doses [5–7]. A landmark study by Darby et al. found a linear dose–response relationship between major cardiac events and mean heart dose, with a 7.4% increase in major coronary events per Gray (Gy). No significant threshold was identified. [7]. Today, advancements in linear accelerator technology and radiotherapy planning have resulted in a decrease in the mean heart dose. In Taylor et al.'s review of cardiac doses for irradiations between 2003 and 2013, the mean heart dose was 5.4 Gy [8]. In the review of Drost et al., which included 99 studies involving breast irradiation between 2014 and 2017, the mean cardiac dose obtained without respiratory control was 4.7 Gy [9].

In the early 2000s, the field of radiation therapy (RT) began to focus on various technological strategies to reduce cardiac dose [10, 11]. Today, the most studied and widely used techniques for reducing cardiac doses are voluntary deep-inspiratory breath-hold (v_DIBH) and deep-inspiratory breath-hold with the active breathing coordinator™ (ABC_DIBH). These techniques allow irradiation when the heart and left anterior descending (LAD) artery are located in the farthest position from the beam field by taking advantage of the physiological respiratory movement pushing the heart into the deep position in the thorax [12].

Although the advantages of respiratory motion management techniques in RT have been demonstrated in many studies, it is not possible for every patient to reach this technique in practice and use it for every breast cancer patient [13–15]. It requires patient education and can increase the workload of the clinic. Since the system setup is complex and the treatment delivery takes longer, the technique is time-consuming, and constant patient cooperation is needed [16]. In addition, due to this more costly technique, there may be problems and delays in the patient appointment system in routine clinical practice.

Dosimetric researches on respiratory-controlled RT is generally based on a dosimetric comparison of breath-hold and free-breathing (FB) techniques and demonstrates the cardiac dose advantage over the free-breathing technique [17–19]. However, the time-consuming nature of the procedure and the high patient load in some centers may make it impractical to provide respiratory-controlled RT to every breast cancer patient. Considering financial and time-related losses, the issue of patient selection for cardiac-protective techniques becomes significant. The present study aimed to examine the presence of some predictive factors and cutoff points to quickly determine which patients would be the main candidates for radiotherapy techniques that protect the heart.

## Material and methods

### Patient selection

Patients with left breast cancer between the ages of 18–80 who received BSC or mastectomy, did not receive RT previously, and had RT to the left breast with or without nodal irradiation were included in the study. The patients included in the study had no previously known heart disease, lung disease, or thoracic anatomical disorders (e.g., pectus excavatum, pectus carinatum).

### Simulation and treatment planning

All patients were immobilized in the supine position with both arms up and head turned to the opposite breast position using the CIVCO C-Qual™ breast board. Computed tomography (CT) images were taken with 3 mm slice thickness throughout the entire neck and thorax regions. In all cases, Clinical Target Volume-breast (CTV), Planning Target Volume-breast (PTV), and non-cardiac OAR (Organs at risk) (lungs, esophagus, spinal cord, right breast) were delineated by the radiation oncologist according to the RTOG (Radiation therapy oncology group) breast contouring atlas [20]. The contoured images were transferred to the TPS (Treatment planning system) of Monaco 5.1 (Elekta AB PUBL, Stockholm, Sweden). A total dose of 50 Gy (2 Gy/fraction/day) was prescribed to the PTV breast using 6 MV-X energy, and a boost dose of 10 Gy (2 Gy/fraction/day) was prescribed to the PTV-primary tumor bed. During the planning, the tangential breast fields with a single isocenter were constructed and structured with the Field-in-field (FIF) planning technique based on the dose limits in the clinical protocol. The plan acceptability criteria were kept as follows: 95% of the breast planning target volume (PTV-breast) or 90% of PTV-chest wall should be covered by at least 90% isodose and volume covered by 107% isodose, should be less than 2 cc.

### Dosimetric and volumetric parameters

Dose-volume histograms (DVHs) were generated according to the QUANTEC (Quantitative Analysis of Normal Tissue Effects in the Clinic) dose limitations. While the RT plans were grouped according to heart doses, the dose-volume parameters of the plans were recorded in the statistical program. The recorded dose-volume parameters were PTV volume, PTV min, max, and mean doses; left lung volume, lung min, max, and mean doses; and lung V5, V10, and V20 values, heart volume, heart min, max, mean, and V10, V25, V35 values, medulla spinalis (MS) volume, and MS min, max, mean doses. On the other hand, plans with a mean heart dose < 4.7 Gy and a heart V25 ≤ 10% were grouped as plans suitable for the FB technique, and plans exceeding these dose limits were grouped as DIBH candidate plans. It was planned to include PTV, left lung, and heart volumes in statistical analysis to predict the appropriateness of cardiac tolerance doses. In addition, heart/PTV, lung/PTV, and lung/heart volume ratios were calculated to make a standard comparison independent of the patient's height, weight, and PTV volume. The data were then processed in SPSS to determine the variables that would be useful for predicting whether or not the plans would be able to meet QUANTEC dose constraints.

### Statistical analysis

Statistical analyses were performed with SPSS 22.0 (SPSS Inc., IBM Corp., Armonk, NY). The variables were investigated using visual (histograms, probability plots) and analytical methods (Kolmogorov–Siminov/Shapiro–Wilk's test) to determine whether or not they are normally distributed. Since the variables were not normally distributed, descriptive analyses were presented using median and minimum–maximum values. The left lung volume, heart volume, PTV volume, lung/PTV volume rate, heart/PTV volume rate, and lung/heart volume rate in predicting the suitability of the RT plan in terms of cardiac tolerance doses were analyzed using ROC (Receiver Operating Characteristics) curve analysis. When a statistically significant cutoff value was observed, the sensitivity, specificity, and positive and negative predictive values were presented. As the data were not normally distributed, Kruskal–Wallis tests were performed on the groups. A *p* value of less than 0.05 was considered statistically significant.

## Results

A total of 114 female patients with left breast cancer were included in the study. According to the AJCC 2017 (AJCC 8th edition) staging system, the stages of patients who underwent BCS and mastectomy were stage 0 in 1.8% (1) and 1.7% (1), stage 1 in 50% (28) and 50% (29), stage 2 in 39.3% (22) and 20.7% (12), stage 3 in 7.1% (4) and 25.9% (15), and stage 4 in 1.8% (1) and 1.7% (1), respectively. Table 1 summarizes the other descriptive statistics of the data.

**Table 1:** Descriptive statistics of the data

Factors	BCS Group (*n* = 56)			Mastectomy Group (*n* = 58)		
	Median	Minimum	Maximum	Median	Minimum	Maximum
Age	54	34	79	53	34	78
PTV volume (cc)	1104.0	417.7	2273.0	633.8	188.4	1239.0
PTV min (Gy)	33.3	9.5	50.4	36.0	13.67	50.4
PTV max (Gy)	62.5	51.6	69.0	55.7	51.5	65.3
PTV mean (Gy)	51.8	41.5	62.1	51.3	48.9	57.2
Left lung volume (cc)	1142.5	759.7	1564.3	1130.2	740.7	2241.0
Lung min (Gy)	0.5	0.1	2.7	0.3	0.1	3.4
Lung max (Gy)	54.7	47.3	66.4	53.9	49.8	66.6
Lung mean (Gy)	12.4	5.1	20.8	15.5	3.8	51.1
Lung V5 (%)	40.0	12.1	64.3	44.0	11.0	65.2
Lung V10 (%)	30.0	11.3	57.6	37.0	8.5	50.2
Lung V20 (%)	24.4	7.8	49.2	30	5.7	42.8
Heart volume (cc)	674.1	466.3	1064.0	614.3	441.1	848.1
Heart min (Gy)	0.8	0.3	45.0	0.4	0.1	40.0
Heart max (Gy)	51.3	12.5	58.5	51.4	5.5	54.6
Heart mean (Gy)	5.0	2.1	10.2	6.4	2.5	16.2
Heart V10 (%)	10.0	2.2	37.0	13.1	6.0	38.0
Heart V25 (%)	6.6	0.9	50.0	10.0	3.5	32.0
Heart V35 (%)	4.6	0.5	30.0	8.0	1.9	28.0

BCS: Breast-conserving surgery, PTV: Planning target volume, n: number of patients, Gy: Gray, cc: cubic centimeter, max: maximum, min: minimum

**Fig. 1:**
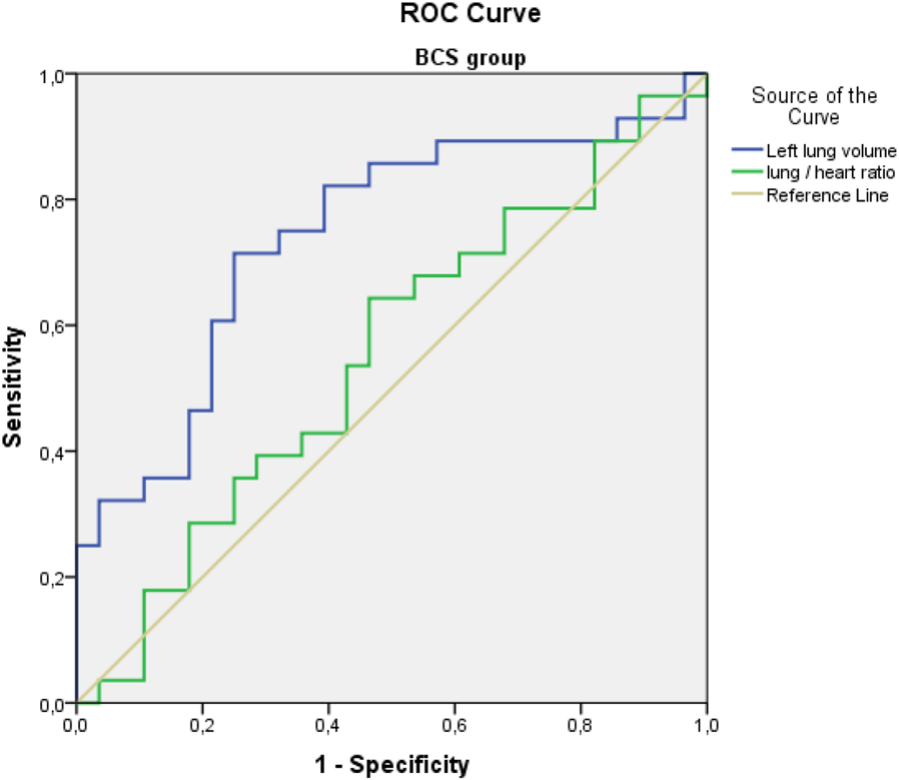
ROC analysis curves of the lung/heart volume ratio and left lung volume for the breast-conserving surgery group

**Fig. 2:**
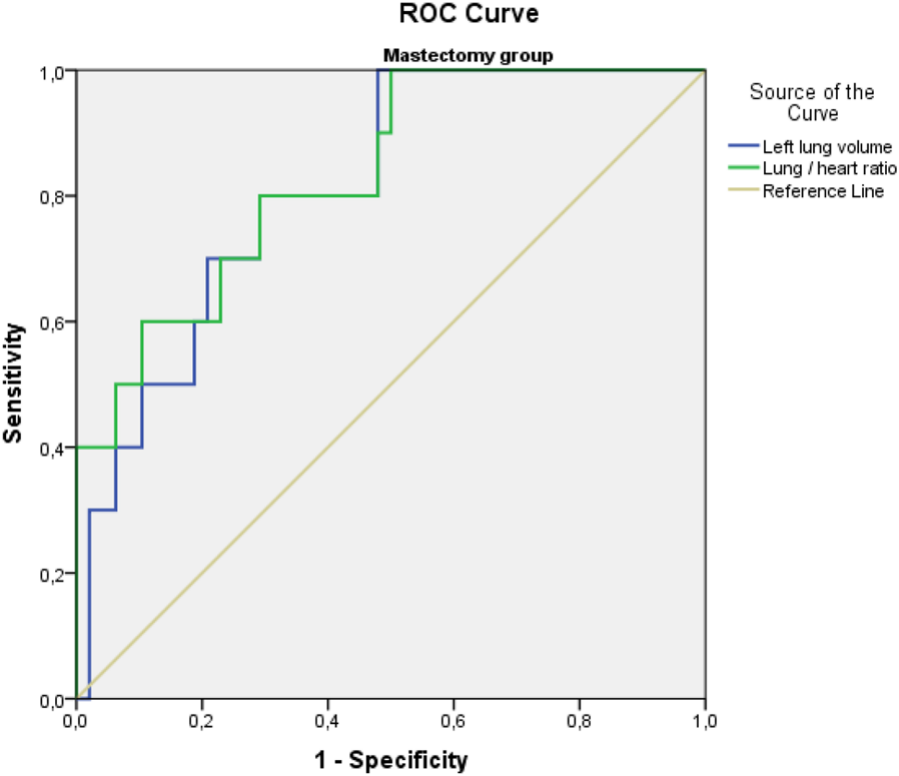
ROC analysis curves of the lung/heart volume ratio and left lung volume for the mastectomy group

Parameters that might affect cardiac doses were evaluated by ROC analysis. Accordingly, when PTV, left lung, heart volumes, lung/heart volume ratio, heart/PTV volume ratio, and lung/PTV volume ratio were evaluated, left lung volume (AUC: 0.72, 95% CI 0.62–0.82, *p* = 0.000) and PTV volume (AUC: 0.66, 95% CI 0.55–0.82, *p* = 0.005) were determined to have a statistically significant impact. When the groups were evaluated separately, only left lung volume (AUC: 0.74, 95% CI 0.61–0.87, *p* = 0.002) was significant in patients who underwent breast-conserving surgery, and in cases with mastectomy, left lung volume (AUC: 0.81, 95% CI 0.69–0.94, *p* = 0.002), and lung/heart volume ratio (AUC: 0.83, 95% CI 0.70–0.96, *p* = 0.001) had a statistically significant effect on the relevance of heart doses (Figs. 1, 2). After the ROC analysis, cutoff points were chosen that could be practical in daily routine with a combination of high sensitivity and specificity. The cutoff point of 1.92 was selected for the lung/heart volume ratio for the mastectomized patients. Furthermore, the cutoff values of 1154 cc and 1208 cc were selected for the left lung volume for the BCS and mastectomized patients, respectively. The sensitivity, specificity, and positive and negative predictive data of the cutoff points are presented in Table 2.

**Table 2:** Sensitivity, specificity, positive, and negative predictive data of the cutoff points according to the groups

Limit value	BCS group^a^				Mastectomy group^a^			
	Sensitivity (%)	Specificity (%)	Positive predictive value (%)	Negative predictive value (%)	Sensitivity (%)	Specificity (%)	Positive predictive value (%)	Negative predictive value (%)
Left lung volume	67.9	75.0	73.1	70.0	80.0	70.8	36.4	94.4
Lung/Heart rate	–	–	–	–	80.0	70.0	36.4	94.4

BCS: Breast-conserving surgery

^a^Cut off points:

Left lung volume 1154 cc for the BCS and 1208 cc for the mastectomy group

Lung/Heart volume ratio: 1.92 for the mastectomy group

The Kruskal–Wallis test was performed to evaluate the effect of the cutoff points found on the dosimetric and volumetric parameters between the groups (Tables 3 and 4). In the dosimetric analysis performed according to the selected cutoff points, mean heart, V10, V25, and V35 values were statistically significantly lower (*p* < 0.05). The median–mean heart dose difference between the groups was 1.5 Gy (*p* < 0.05).

**Table 3:** Dosimetric and volumetric parameters between groups according to left lung volume

	BCS group (*n* = 56)		p*	Mastectomy group (*n* = 58)		
	≤ 1154 cc median (min–max)	> 1154 cc median (min–max)		≤ 1208 cc median (min–max)	> 1208 cc median (min–max)	p*
PTV volume (cc)	1178.2 (450.4–2273.0)	1162.9 (417.7–2060.0)	0.793	659.9 (188.4–1239.0)	474.6 (234.9–1040.8)	0.034
PTV min (Gy)	30.5 (9.5–40.1)	29.2 (10.6–49.7)	0.974	36.0 (13.6–50.4)	36.0 (20.1–50.4)	0.486
PTV max (Gy)	63.3 (53.8–69.0)	62.2 (51.6–66.6)	0.026	55.7 (51.5–64.9)	55.5 (53.2–65.3)	0.712
PTV mean (Gy)	52.3 (49.9–55.7)	51.7 (41.5–62.1)	0.206	51.1 (48.9–53.4)	51.4 (49.0–57.2)	0.100
Left lung volume (cc)	1056.5 (759.7–1164.0)	1244.4 (1171.5–1564.3)	0.000	1047.4 (740.7–1205.0)	1346.4 (1211.1–2241.0)	0.000
Lung min (Gy)	0.5 (0.1–1.0)	0.6 (0.1–2.7)	0.941	0.3 (0.1–3.4)	0.2 (0.0–0.9)	0.019
Lung max (Gy)	55.4 (48.7–66.4)	53.0 (47.3–61.4)	0.054	53.6 (50.1–66.6)	54.4 (49.8–58.1)	0.061
Lung mean (Gy)	13.2 (5.1–20.8)	11.7 (5.8–17.8)	0.511	15.6 (7.9–20.6)	15.3 (3.8–51.1)	0.911
Lung V5 (%)	40.2 (12.1–64.3)	36.3 (20.5–60.8)	0.651	44.0 (11.0–65.2)	41.1 (12.7–53.6)	0.100
Lung V10 (%)	30.5 (11.3–57.6)	29.7 (12.8–49.9)	0.628	37.0 (9.0–50.2)	34.5 (8.5–42.9)	0.335
Lung V20 (%)	25.8 (7.9–49.1)	22.5 (8.78–37.1)	0.501	30.0 (7.0–42.8)	29.0 (5.7–37.0)	0.381
Heart volume (cc)	674.1 (516.4–1064.1)	675.5 (466.3–996.4)	0.588	620.6 (441.1–848.1)	599.3(499.7–821.8)	0.898
Heart min (Gy)	0.8 (0.3–16.9)	0.8 (0.3–45.0)	0.571	0.4 (0.2–40.0)	0.3 (0.1–1.0)	**0.009**
Heart max (Gy)	**52.0 (12.5–58.5)**	**50.0 (42.1–58.0)**	**0.015**	**51.0 (5.55–54.6)**	**51.8 (48.3–53.9)**	0.214
Heart mean (Gy)	**6.2 (2.6–10.2)**	**4.5 (2.1–9.1)**	**0.001**	**7.1 (4.2–916.2)**	**4.5 (2.5–8.0)**	**0.000**
Heart V10 (%)	**12.3 (2.2–37.0)**	**8.8 (4.0–20.0)**	**0.007**	**16.0 (9.5–38.0)**	**11.7 (6.0–20.0)**	**0.006**
Heart V25 (%)	**8.2 (0.1–50.0)**	**5.6 (2.4–18.0)**	**0.020**	**11.0 (5.2–32.0)**	**8.0 (3.5–17.0)**	**0.002**
Heart V35 (%)	**5.6 (0.5–30.0)**	**4.0 (1.7–16.0)**	**0.036**	**8.1 (1.9–28.0)**	**7.0 (2.4–15.0)**	**0.017**

BCS: Breast-conserving surgery, PTV: Planning target volume, n: number of patients, Gy: Gray, cc: cubic centimeter, max: maximum, min: minimum

*Kruskal–Wallis test

Bold values indicate the significant p values (p<0.05)

**Table 4:** Dosimetric and volumetric parameters between groups according to left lung/heart volume ratio only in the mastectomy group (n = 58)

	Left lung/heart volume ratio		p*
	≤ 1.92 median (min–max)	> 1,92 median (min–max)	
PTV volume (cc)	**661.3 (188.4–1239.0)**	**454.0 (234.9–884.6)**	**0.016**
PTV min (Gy)	36.0 (13.6–50.4)	36.0 (23.1–50.4)	0.625
PTV max (Gy)	55.5 (51.5–63.7)	56.2 (54.0–65.3)	0.102
PTV mean (Gy)	**51.1 (48.9–53.4)**	**51.4 (50.0–57.2)**	**0.045**
Left lung volume (cc)	**1055.3 (740.7–1365.2)**	**1318.0 (1021.8–2241.0)**	**0.000**
Lung min (Gy)	**0.3 (0.1–3.4)**	**0.2 (0.1–2.4)**	**0.024**
Lung max (Gy)	**53.2 (49.8–66.6)**	**54.5 (5.6–58.1)**	**0.001**
Lung mean (Gy)	15.8 (8.5–51.1)	14.8 (3.8–19.1)	0.229
Lung V5 (%)	44.0 (22.0–65.2)	40.0 (11.0–62.0)	0.029
Lung V10 (%)	37.0 (9.0–50.2)	33.0 (8.5–50.0)	0.095
Lung V20 (%)	30.0 (7.0–42.8)	28.0 (5.7–37.0)	0.117
Heart volume (cc)	**667.8 (499.6–848.1)**	**576.0 (441.1–821.8)**	**0.004**
Heart min (Gy)	**0.5 (0.2–40.0)**	**0.3 (0.1–4.4)**	**0.002**
Heart max (Gy)	**51.0 (5.5–54.6)**	**52.1 (49.0–53.9)**	**0.027**
Heart mean (Gy)	**7.1 (4.5–16.2)**	**5.6 (2.5–8.3)**	**0.002**
Heart V10 (%)	**16.5 (8.1–39.0)**	**12.0 (6.0–20.0)**	**0.012**
Heart V25 (%)	**10.5 (5.2–32.0)**	**8.5 (3.5–17.0)**	**0.020**
Heart V35 (%)	8.05 (1.9–28.0)	7.5 (2.4–15.0)	0.118

PTV: Planning target volume, n: number of patient, Gy:Gray, cc:cubic cantimeter, max: maximum, min:minimum

* Kruskal–Wallis test

Bold values indicate the significant p values (p<0.05)

## Discussion

Radiation therapy (RT) has a vital role in the treatment of breast cancer, and it is the main component of adjuvant treatment in breast cancer patients. Radiation-induced cardiac damage begins to occur without any threshold point, and there is a 7.4% increase in the risk of major cardiac events with each 1 Gy dose delivered to the heart [7]. Although the main goal is to protect the heart from radiation at the maximum level in breast RT, techniques that protect the heart are equipment-dependent. Furthermore, simulation with the DIBH technique, patient education during simulation, RT planning, and the application take more time than conformal RT with the FB technique. This study aimed to examine the presence of some predictive factors and cutoff points to quickly determine which patients are the main candidates for RT techniques that protect the heart.

Dosimetric studies of respiratory-controlled RT are generally based on a dosimetric comparison of DIBH and FB techniques and a demonstration of cardiac dose advantage with DIBH over FB technique [17–19]. A few dosimetric studies have attempted to identify patients who would benefit more from the DIBH technique [21–25].

The patient's anatomical features affect the results of the planning. Czeremszyn'ska et al. aimed to determine some thresholds of the anatomical characteristics as dosimetric predictors. Among these, body mass index (BMI), cardiac contact distance (CCD), PTV volume, and lung volume in FB were investigated, and it was demonstrated that other anatomical characteristics, except lung volume, can affect dosimetric parameters by 20% or 50% at certain cutoff points. Although the left lung volume increased with the DIBH method, a cutoff point related to the left lung volume could not be determined. In this study, it was stated that there are also patients who would benefit from DIBH below the cutoff points; therefore, they should not be used in practice [22].

One of the parameters investigated to select patients who will benefit maximum from DIBH is maximum heart depth (MHD). With the DIBH method, the heart reaches a deeper position in the thorax and the heart–chest wall distance increases. In the study of Ferdinand et al. a 46.7% reduction (2.01 cm in FB scans vs. 1.07 cm in DIBH scans) (*p* < 0.001) was obtained in MHD with the DIBH technique. As a result, a decrease from 4 to 2.4 Gy was obtained in the mean heart dose and from 12.6 to 8.7 Gy in the mean LAD dose. Ferdinand et al. also reported that there would be a 50% reduction in mean heart dose in patients with DIBH with a difference of > 1 cm in MHD [21]. Taylor et al. revealed an increase of 2.9% in mean heart dose with every 1 cm of MHD [23]. Tanna et al. nominated patients with this depth of > 1 cm for the DIBH technique [24]. Patients with a difference of > 1 cm in MHD with DIBH can be nominated for the DIBH technique, since a significant reduction in mean-heart dose will be achieved.

Another dosimetric predictor reported in the literature is the heart volume in the field (HVIF). A study by Wang et al. indicated that the mean heart dose increases by 0.67 Gy per 1-cc increase in HVIF [25]. In the study of Ferdinand et al., a 73.8% reduction (26.58 ccs in FB scans vs. 7.02 cc in DIBH scans) (*p* < 0.001) was obtained in Heart Volume In Field (HVIF) with the DIBH technique. For Delta HVIF, a 20% reduction in mean heart dose can be achieved with a cutoff value of 6 ccs and a reduction of > 50% with a cutoff value of 13 ccs [21].

All these values mentioned are the factors that would be obtained after the RT fields are located and almost all the plans are made. In our study, however, no additional examination was performed on these parameters, since it was investigated the predictive parameters that could be determined before the planning phase. This condition indicates that our study has practical results compared to other studies. Table 5 shows the comparison of the technique, the factors examined, and the cutoff values for our study and other studies.

**Table 5:** Comparison of the technique, the factors examined and the cutoff values for our study and other studies

Study	Teknik	İncelenen faktörler	Bulunan cut-off değerler
Ferdinand et al. [21]	Dosimetric results of free breathing (FB) vs. deep inspiration breath hold (DIBH) techniques are compared	(1) Heart volume (HV) (2) Lung volume (LV) (3) Heart chest wall length (HCWL) (4) Heart height (HH) (5) Chest separation (CS) (6) Chest depth (CD) (7) Heart chest wall distance (HCWD) (8) Maximum heart depth (MHD) (9) Heart volume in field (HVIF) (10) Lung ortogonal distance (LOD) (11) Central lung distance (CLD)	Maksimum heart depth = 7 mm ΔHeart volume in field = 6 cc
Czeremszyn´ska et al. [22]	Dosimetric results of FB vs. DIBH techniques are compared	(1) Body mass index (BMI) (2) Age (3) Planning target volume (PTV) (4) Cardiac contact distance (CCD) (5) Lung volume at FB	For ΔMHD, ΔV20 Heart and ΔLADmax For 20% improvement, in order; In the BMİ 22.3, 22.3, 24.8 In the CCD 2.9, 2.9, 3.8 cm In the PTV volume 577, 445 cc For 50% improvement, in order; In the BMİ 27.6, 22.3, 26.0 In the CCD 5.7, 2.9, 3.0 cm In the PTV volume 703, 445 cc
Taylor et al. [23]	To assess the value of MHD in predicting the dose and biologically effective dose (BED) to the heart and the left anterior descending (LAD)	(1) Mean dose and BED to the heart, (2) Mean dose and BED to the LAD coronary artery, (3) MHD, (4) Position of the CT slice showing the maximum area of the irradiated heart relative to the mid-plane slice, and (5) Sternal and contralateral breast thickness (measures of body fat)	Every 1-cm increase in MHD
Tanna et al. [24]	Comparing four methods of patient selection for FB or DIBH	(1) FB scan on all patients, selecting DIBH technique for mean heart dose ≥ 3 Gy; (2) Selective DIBH for those with MHD on FB scan ≥ 1 cm; (3) Use of an ‘upfront selection process’ using tumor bed position as initial selection and measurement of MHD on those not selected upfront; (4) DIBH on all	‘Upfront selection process’ MHD
Wang et al. [25]	The automated treatment planning process is investigated		The heart volume within the radiation field (heart V50 > 10 cm3)
Our study	Before starting the planning, factors that will predict the choice of technique are investigated	(1) PTV volume (2) Left lung volume (3) Heart volume (4) Left lung/heart volume ratio (5) Heart/PTV volume ratio (6) Left lung/PTV volume ratio	Lung/heart volume ratio = 1.92 Left lung volume = 1154 for BCS patients and 1208 cc for mastectomy patients

Even if RT is planned with the DIBH technique in patients with left breast cancer, it may not be possible for all of these patients to benefit from or complete the treatment with this technique. In the study of Czeremszyn'ska et al. only 63% (19/30) of the patients who achieved 20% dosimetric advantage with the DIBH technique could complete their treatment with DIBH, since they could not keep their breath efficiently throughout the whole treatment course. Therefore, this study indicates that about 20% of breast cancer patients would not comply with this technique [22]. According to the 7 mm and 6 cc cutoff values in the MHD and HVIF factors predicted by Ferdinand et al. it was determined that 9 (29%) of 31 patients would benefit less from the DIBH technique [21]. The present study determined that cardiac doses would remain within the tolerance limits in 19 (73%) of 26 patients whose cutoff value was higher than the cutoff value, considering only the 1154 cc cutoff point in the left lung volume before planning for BCS patients. In patients with mastectomy, if cutoff points of 1.92 cc were used for lung/heart volume ratio and 1208 cc for left lung volume, for both cutoff values, it was determined that 8 (38%) of 22 patients would currently have a heart dose of 5 Gy or less with the conventional technique, and they would benefit less from the DIBH technique. If these two cutoff points were used simultaneously, heart doses would remain within the desired tolerance limits in 7 (39%) of 18 patients.

A comparison of the cost-effectiveness of DIBH is still incomplete and will be investigated shortly. Compared to other techniques, the conventional technique is still low in cost [26].

Our study has some strengths and weaknesses. One of its strengths is that the parameters used in the study are objective and simple volumetric parameters that can be obtained without losing time in RT planning. Undoubtedly, it is possible to reduce the heart dose to a certain extent with the breath hold technique. However, this technique may not be available in some hospitals, and directing the patient geographically to the center, where the respiratory-controlled RT technique that is located may increase the waiting time and lead to loss of time and financial losses. Besides, using these cutoff values can enable a quick selection of patients who will benefit from this technique in centers with high density due to this time-consuming technique. Moreover, it can be used as a rapid selection method for immediate referral to patients at high risk for cardiac dose. One of the weaknesses of our study is the retrospective nature of the design. Due to the retrospective design, there was no chance to compare v_DIBH with the normal technique as in the UK HeartSpare study [12]. Furthermore, due to the limited number of patients, patients who received breast radiotherapy, chest wall radiotherapy, and RT to peripheral lymph nodes were examined together. In the study of Ferdinand et al., no dosimetric difference was determined between the patients whose regional lymph nodes were irradiated and those whose regional lymph nodes were not irradiated [21]. In addition, since the LAD artery is not contoured in the clinical routine, it has not been provided how the cutoff points determined will affect the LAD doses. Considering all this information, the RT planning technique for breast cancer patients should be selected according to all the advantages and disadvantages of existing data.

## Conclusion

The cutoff value of 1154 cc in left lung volume for patients with BCS in left breast radiotherapy and 1.92 and 1208 cc cutoff points in the lung/heart volume ratio for patients with mastectomy can be used to predict whether RT plans will meet QUANTEC heart dose limits. Evaluating only these few cutoff points before planning makes it possible to eliminate 70% of patients with BCS and 40% of patients with mastectomy from respiratory-controlled methods, which require time and effort. Patients with a lung volume and a lung/heart volume ratio lower than the cutoff values are prime candidates for heart-sparing techniques.

## Data Availability

The present data are summarized in this paper. The complete data set can be retrieved from the authors upon formal request from interested readers.

## References

[1] Darby S, McGale P, Correa C, Taylor C, Arriagada R, Early Breast Cancer Trialists’ Collaborative Group (EBCTCG) (2011). Effect of radiotherapy after breast-conserving surgery on 10-year recurrence and 15-year breast cancer death: meta-analysis of individual patient data for 10,801 women in 17 randomised trials. Lancet.

[2] Sedlmayer F, Sautter-Bihl ML, Budach W, Dunst J, Fastner G, Feyer P (2013). DEGRO practical guidelines: radiotherapy of breast cancer I: Radiotherapy following breast conserving therapy for invasive breast cancer. Strahlentherapie Und Onkol.

[3] Fisher B, Anderson S, Bryant J, Margolese RG, Deutsch M, Fisher ER (2002). Twenty-year follow-up of a randomized trial comparing total mastectomy, lumpectomy, and lumpectomy plus irradiation for the treatment of invasive breast cancer. N Engl J Med.

[4] Abe O, Abe R, Enomoto K, Kikuchi K, Koyama H, Masuda H (2005). Effects of radiotherapy and of differences in the extent of surgery for early breast cancer on local recurrence and 15-year survival: An overview of the randomised trials. Lancet.

[5] Gagliardi G, Lax I, Ottolenghi A, Rutqvist LE (1996). Long-term cardiac mortality after radiotherapy of breast cancer–application of the relative seriality model. Br J Radiol.

[6] Paszat LF, Vallis KA, Benk VMA, Groome PA, Mackillop WJ, Wielgosz A (2007). A population-based case-cohort study of the risk of myocardial infarction following radiation therapy for breast cancer. Radiother Oncol.

[7] Darby SC, Ewertz M, McGale P, Bennet AM, Blom-Goldman U, Brønnum D (2013). Risk of ischemic heart disease in women after radiotherapy for breast cancer. N Engl J Med.

[8] Taylor CW, Zhe W, Macaulay E, Jagsi R, Duane F, Darby SC (2015). Exposure of the heart in breast cancer radiation therapy: a systematic review of heart doses published during 2003 to 2013. Int J Radiat Oncol Biol Phys.

[9] Drost L, Yee C, Lam H, Zhang L, Wronski M, McCann C (2018). A systematic review of heart dose in breast radiotherapy. Clin Breast Cancer.

[10] Raj KA, Evans ES, Prosnitz RG, Quaranta BP, Hardenbergh PH, Hollis DR (2006). Is there an increased risk of local recurrence under the heart block in patients with left-sided breast cancer?. Cancer J.

[11] Korreman SS, Pedersen AN, Nøttrup TJ, Specht L, Nyström H (2005). Breathing adapted radiotherapy for breast cancer: comparison of free breathing gating with the breath-hold technique. Radiother Oncol.

[12] Bartlett FR, Colgan RM, Carr K, Donovan EM, McNair HA, Locke I (2013). The UK HeartSpare Study: randomised evaluation of voluntary deep-inspiratory breath-hold in women undergoing breast radiotherapy. Radiother Oncol.

[13] Lin A, Sharieff W, Juhasz J, Whelan T, Kim DH (2017). The benefit of deep inspiration breath hold: evaluating cardiac radiation exposure in patients after mastectomy and after breast-conserving surgery. Breast Cancer.

[14] Lai J, Hu S, Luo Y, Zheng R, Zhu Q, Chen P (2020). Meta-analysis of deep inspiration breath hold (DIBH) versus free breathing (FB) in postoperative radiotherapy for left-side breast cancer. Breast Cancer.

[15] Latty D, Stuart KE, Wang W, Ahern V (2015). Review of deep inspiration breath-hold techniques for the treatment of breast cancer. J Med Radiat Sci.

[16] Haji G, Nabizade U, Kazimov K, Guliyeva N, Isayev I (2019). Liver dose reduction by deep inspiration breath hold technique in right-sided breast irradiation. Radiat Oncol J.

[17] Dell’Oro M, Giles E, Sharkey A, Borg M, Connell C, Bezak E (2019). A retrospective dosimetric study of radiotherapy patients with left-sided breast cancer; patient selection criteria for deep inspiration breath hold technique. Cancers.

[18] Xin X, Li J, Zhao Y, Wang P, Tang B, Yao X (2021). Retrospective study on left-sided breast radiotherapy: dosimetric results and correlation with physical factors for free breathing and breath hold irradiation techniques. Technol Cancer Res Treat.

[19] Gaál S, Kahán Z, Paczona V, Kószó R, Drencsényi R, Szabó J (2021). Deep-inspirational breath-hold (DIBH) technique in left-sided breast cancer: various aspects of clinical utility. Radiat Oncol.

[20] RADCOMP Breast Atlas n.d. https://www.nrgoncology.org/About-Us/Center-for-Innovation-in-Radiation-Oncology/Breast-Cancer/RADCOMP-Breast-Atlas Accessed 6 November 2022.

[21] Ferdinand S, Mondal M, Mallik S, Goswami J, Das S, Manir KS (2021). Dosimetric analysis of Deep Inspiratory Breath-hold technique (DIBH) in left-sided breast cancer radiotherapy and evaluation of pre-treatment predictors of cardiac doses for guiding patient selection for DIBH. Tech Innov Patient Support Radiat Oncol.

[22] Czeremszyńska B, Drozda S, Górzyński M, Kępka L (2017). Selection of patients with left breast cancer for deep-inspiration breath-hold radiotherapy technique: results of a prospective study. Reports Pract Oncol Radiother.

[23] Taylor CW, McGale P, Povall JM, Thomas E, Kumar S, Dodwell D (2009). Estimating cardiac exposure from breast cancer radiotherapy in clinical practice. Int J Radiat Oncol Biol Phys.

[24] Tanna N, McLauchlan R, Karis S, Welgemoed C, Gujral DM, Cleator SJ (2017). Assessment of upfront selection criteria to prioritise patients for breath-hold left-sided breast radiotherapy. Clin Oncol (R Coll Radiol).

[25] Wang W, Purdie TG, Rahman M, Marshall A, Liu FF, Fyles A (2012). Rapid automated treatment planning process to select breast cancer patients for active breathing control to achieve cardiac dose reduction. Int J Radiat Oncol Biol Phys.

[26] Xie Y, Guo B, Zhang R (2021). Cost-effectiveness analysis of radiotherapy techniques for whole breast irradiation. PLoS ONE.

